# The Role of GABAergic Inhibition in Ocular Dominance Plasticity

**DOI:** 10.1155/2011/391763

**Published:** 2011-08-02

**Authors:** J. Alexander Heimel, Daniëlle van Versendaal, Christiaan N. Levelt

**Affiliations:** Department of Molecular Visual Plasticity, Netherlands Institute for Neuroscience, Royal Netherlands Academy of Arts and Sciences, Meibergdreef 47, 1105BA Amsterdam, The Netherlands

## Abstract

During the last decade, we have gained much insight into the mechanisms that open and close a sensitive period of plasticity in the visual cortex. This brings the hope that novel treatments can be developed for brain injuries requiring renewed plasticity potential and neurodevelopmental brain disorders caused by defective synaptic plasticity. One of the central mechanisms responsible for opening the sensitive period is the maturation of inhibitory innervation. Many molecular and cellular events have been identified that drive this developmental process, including signaling through BDNF and IGF-1, transcriptional control by OTX2, maturation of the extracellular matrix, and GABA-regulated inhibitory synapse formation. The mechanisms through which the development of inhibitory innervation triggers and potentially closes the sensitive period may involve plasticity of inhibitory inputs or permissive regulation of excitatory synapse plasticity. Here, we discuss the current state of knowledge in the field and open questions to be addressed.

## 1. Sensitive Periods of Plasticity

Many things can be learned more easily during childhood than in adulthood, including speaking a new language, playing an instrument, or performing a sport. This is the consequence of how our brain develops. It seems to make sense to learn these skills in a rather permanent way when we are young so that we can take advantage of them when we are adults. This is not only true for learning skills or facts but reflects a general property of brain development where periods of enhanced experience-dependent plasticity in different cortical and subcortical brain regions are essential for achieving functional and reliable connectivity between brain areas. During the last decade, it has become clear that specific molecular and cellular mechanisms are in place that regulate the onset and offset of these sensitive periods [1], indicating that they are not simply the consequence of the brain regions involved becoming optimized but actively regulated periods of enhanced plasticity. Sensitive periods are not only essential for normal brain development, they are also protective in cases of brain damage during childhood. In the young brain, cortical areas are not yet fully committed to specific tasks and damage can still be compensated for by other brain areas taking over the lost functionality [2]. But sensitive periods can also cause important problems. If plasticity does not occur in a proper fashion during these periods, lifelong problems may occur. This can occur if the provided inputs are inadequate. The best studied example is the development of amblyopia (lazy eye) which happens when one eye does not function well during development, driving plasticity in the visual cortex to respond less accurately to inputs from this eye [3]. Flawed plasticity may also occur due to genetic deficits in plasticity mechanisms as is the case in neurodevelopmental disorders such as mental retardation or autism [4]. 

## 2. Ocular Dominance Plasticity

In order to be able to treat such disorders in the future and to develop approaches to increase adult plasticity for treating brain damage, extensive research on the mechanisms opening and closing sensitive periods has been performed. Plasticity in the visual cortex has been the most used model to address this. The primary visual cortex (V1) receives inputs conveying information from both eyes, and neurons in V1 have a preference for inputs from one of the eyes [5]. This so-called ocular dominance (OD) can change when during development input from one eye is less reliable than that from the other, for example due to refractive error, cataract or misalignment of the eyes, or when one eye is closed (monocular deprivation, MD) under experimental conditions [6]. This will result in an OD shift towards the more reliable eye which is accompanied by extensive rewiring of thalamocortical and intracortical connections [7–10]. OD plasticity has a well-defined sensitive period. The timing of this sensitive period differs per species, occurring during the 4th and 5th postnatal week in rodents [11, 12], between 1–3 months in cats [13], and between 6 months and 8 years in humans [14]. However, the changes that occur with OD plasticity are very similar between species and vision improves significantly during the sensitive period in all species tested. While traditionally, cats and monkeys were used for studying visual plasticity, we have seen a switch to the use of rodents for these studies. Although their visual acuity is significantly lower than that of cats and monkeys [11], molecular analysis and modification and in vivo imaging techniques are much more feasible in rodents. This switch of species for studying sensitive period regulation has been a fruitful endeavor. It has led to the discovery of various important regulatory mechanisms that underlie the opening and closure of the sensitive period of OD plasticity. Of particular interest are the findings that the development of inhibitory innervation is essential for sensitive period onset [15] and that the mature extracellular matrix [16] and epigenetic transcriptional regulation [17] are involved in sensitive period offset. 

## 3. Maturation of Inhibition Initiates Sensitive Period of OD Plasticity

The initial evidence supporting the involvement of the inhibitory system in sensitive period onset was the discovery that in mice in which GABA synthesis is reduced due to the deletion of the glutamic acid decarboxylase GAD65, no sensitive period plasticity can be induced until the moment that they are treated with the GABA(A) receptor agonist benzodiazepine [15]. Interestingly, it does not matter whether they are injected during development or in adulthood. Next, it was shown that also in normal mice, benzodiazepines can induce a precocious sensitive period when administered several days before normal sensitive period onset [18]. Compatible observations were made in transgenic animals overexpressing the neural growth factor BDNF [19, 20]. In these animals, the development of inhibitory boutons occurs several days earlier than in control animals and is accompanied by the early onset of the sensitive period. Not only plasticity can be induced earlier, also the development of high visual acuity is accelerated. BDNF is a protein whose expression and release are regulated by neuronal activity [21]. In animals that are reared in the dark, BDNF expression in the visual cortex is reduced and the sensitive period is delayed [12]. Interestingly, in transgenic animals overexpressing BDNF, dark rearing did not delay the onset of the sensitive period nor did it prevent the development of high acuity vision [22], suggesting that BDNF is one of the main factors driving the onset of the sensitive period through spurring the development of inhibitory innervation. More recently, it was found that insulin growth factor-1 (IGF-1) has similar effects as BDNF, accelerating the development of inhibitory synapses, the onset of the sensitive period and the increase of visual acuity [23]. Previous studies demonstrated that IGF-1 stimulates the production of BDNF [24]. IGF-1 may thus act upstream of BDNF during the development of inhibitory innervation and the onset of the sensitive period.

## 4. Parvalbumin Expressing Basket Cells and Sensitive Period Onset

In order to understand the mechanism through which inhibition may initiate the sensitive period, it is important to know which interneuron subtypes are involved. Most evidence points towards the involvement of fast-spiking Parvalbumin (PV) expressing basket cells. These interneurons innervate excitatory neurons [25], each other [26] and other types of interneurons through synaptic boutons localized predominantly on their cell soma and proximal dendrites. PV expressing basket cells fire through trains of high-frequency nonadapting spikes [27]. Their connectivity and synaptic properties put them in the ideal situation to detect and stimulate neuronal synchrony with high speed and precision [26]. In line with these properties, the postsynaptic GABA(A)receptors (GABAAR) they synapse onto predominantly utilize GABAAR alpha1 subunits [28], which form GABAAR with the fastest decay times [29]. In line with the involvement of PV basket cells driving sensitive period onset, benzodiazepines cannot induce a precocious sensitive period in mouse mutants in which the GABAAR alpha1 subunit has been rendered insensitive to benzodiazepines [30]. Although this convincingly shows that inhibitory synapses containing the GABAAR alpha1 subunit are important for initiating the sensitive period, this does not mean that any specific properties of this subunit, such as the fast decay time, are important. Moreover it is important to realize that the GABAARalpha1 subunit is by far the most abundant subunit in the neocortex and is not exclusive to synapses formed by PV basket cells [28, 31]. However, additional evidence for the involvement of PV basket cells in initiating the sensitive period comes from the fascinating finding that the transcription factor OTX2 which is transcribed and translated in the retina is transported transsynaptically to V1 where it is taken up predominantly by PV basket cells and a small percentage of other interneuron subtypes where it stimulates their development [32]. In the absence of OTX2 in the retina, the sensitive period does not start, but can be initiated by supplying OTX2 protein directly to the visual cortex.

## 5. GABA-Mediated Inhibitory Synapse Formation

The formation of inhibitory inputs by PV basket cells onto the somata of excitatory neurons is strongly influenced by neuronal activity around the start of the sensitive period. This development of perisomatic inhibitory innervation can be replicated in cortical slice cultures. Using cultures from transgenic mice expressing GFP in PV interneurons it was found that innervation by PV interneurons was only reduced when activity was inhibited by TTX between p18 and p24, but not when this was done at later stages [33]. Similarly, when TTX is injected in one eye between p20 and p24, in vivo development of perisomatic synapses formed by PV interneurons is reduced, while TTX injection at later stages does not have any effects [33]. Interestingly, it seems that inhibitory transmission itself is what stimulates the formation of inhibitory synapses. This was most convincingly shown by knocking out the *Gad-1* gene (which encodes the rate limiting glutamate decarboxylase GAD67, which is responsible for 90% of GABA synthesis) in individual neurons in the visual cortex, which results in reduced synapse formation and axon branching [34]. Overexpression of GAD67, in contrast, stimulates the formation of perisomatic synapses by PV interneurons. The reduced formation of perisomatic inhibitory synapses can be rescued by inhibition of the GABA reuptake protein GAT-1 effectively increasing GABA levels and partially by enhancing inhibition with benzodiazepines. 

Such a positive feedback loop seems to fit nicely with other findings. For example, as explained above, in mice deficient for GAD65, the sensitive period does not start until inhibitory inputs are strengthened with benzodiazepines [15]. However, when this treatment is stopped, it is not possible to repeat the induction of a sensitive period with benzodiazepines. This indicates that after the first treatment, inhibitory innervation has matured in an irreversible way. A positive feedback loop may well explain this. Also, it was found that expression of polysialic acid (PSA) bound to NCAM-1 in the visual cortex holds off the formation of perisomatic innervation by PV interneurons, probably through interactions with NCAM-1 or other adhesion molecules [35]. Premature removal of PSA initiates a precocious sensitive period. Interestingly, when an early sensitive period is induced with benzodiazepines, this results in a spontaneous reduction of PSA showing that events that increase GABAergic innervation accelerate other events that also enhance GABAergic innervation, thus supporting a positive feedback mechanism [35].

## 6. Inhibition and Closure of the Sensitive Period

**Figure 1:**
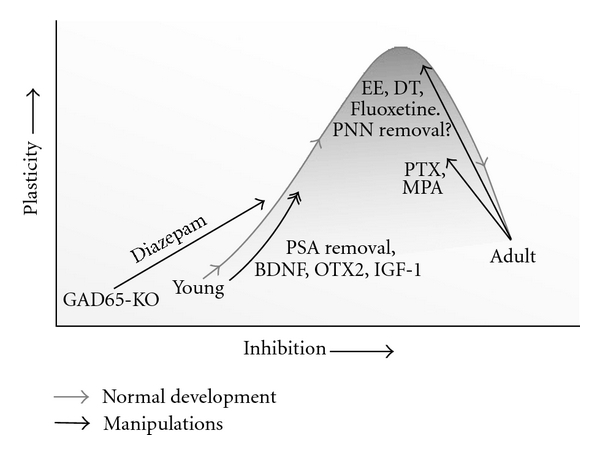
Relationship of inhibition and plasticity during the critical period. Gray line depicts maturation of inhibition and the increase and decline of potential for plasticity during normal development. Black lines show experimental manipulations by which the level of plasticity or inhibition has been artificially altered. EE is environmental enrichment, DT is dark treatment, PNN is perioneuronal net. All manipulations are infusions or injections of substances, except for BDNF which has been overexpressed. See text for references.

Evidence for increased inhibitory inputs being central to the initiation of the sensitive period of OD plasticity is very convincing (Figure 1). It is less clear whether continued maturation of inhibitory innervation is what also closes the sensitive period. In fact, quite some evidence accumulated suggested that other mechanisms are involved in sensitive period offset. Epigenetic regulation of gene transcription for example has been found to close the sensitive period of OD plasticity, while interfering with this process by treating animals with the histone deacetalyse inhibitor Trichostatin reopens a sensitive period [17]. Moreover, there is evidence to suggest that limitation of structural plasticity is what ends the sensitive period. For example, in the absence of Nogo-66 receptor, a receptor for the myelin-based factor Nogo which inhibits axon outgrowth, animals show continued plasticity in the visual cortex into adulthood [36]. Also the extracellular matrix (ECM), which was found to inhibit axon growth after spinal cord injuries [37], forms a barrier for adult cortical plasticity. Dissolving the extracellular matrix in V1 using the enzyme chondroitinase reinstates the plasticity potential in the visual cortex [16], possibly by removing a physical barrier for axon- or spine growth and retraction, or by removing ECM associated factors that limit neurite growth such as semaphorins [38]. In line with this finding is that activation of Plasmin, a protease involved in ECM degradation, also increases spine motility in cortical slices [39], while in the absence of its activator, TPA, OD plasticity is defective [40].

However, the interpretation of how the ECM limits adult cortical plasticity may have to be adjusted based on novel evidence [41]. While the ECM is present throughout the entire brain, it forms dense structures known as perineuronal nets predominantly around PV interneurons [42]. The formation of these perineuronal nets depends on the link protein Ctrl1. In mice deficient for this protein, perineuronal nets do not form while the ECM otherwise looks normal [41]. Interestingly, OD plasticity in Ctrl1-deficient mice can still be induced effectively in adulthood, indicating that it is PV interneuron function that is crucial for sensitive period offset rather than a general restriction of structural plasticity. An interesting possibility is that the perineuronal nets are important for PV interneurons to bind and take up the transcription factor OTX2, which as described above, is essential for their maturation.

What other evidence is there to support the notion that mature levels of inhibitory inputs restrict adult cortical plasticity? Recent studies have shown that when adult rats are monocularly deprived when housed in an enriched environment, OD plasticity can be induced more effectively than in normally reared rats [43]. Histological and molecular analyses of the visual cortices of these animals revealed that this was accompanied by a decrease in perineuronal net densities, expression of the vesicular GABA transporter (VGAT), and intracortical GABA levels. The concurrent treatment of such animals with benzodiazepines blocked the enrichment-induced plasticity potential, suggesting that reduced levels of inhibition indeed represents the underlying mechanisms. Decreased intracortical GABA levels and increased adult plasticity that could be blocked with benzodiazepine administration was also observed in animals treated with the selective serotonin reuptake inhibitor Fluoxetine [44]. More direct evidence for increased levels of inhibition reducing adult cortical plasticity comes from the finding that treating adult rats with the GABA antagonist picrotoxin or GABA synthesis inhibitor MPA during a period of MD, increases OD plasticity [45]. However, while this treatment increases adult plasticity, it does not allow the induction of a full OD shift as observed during the sensitive period (Figure 1). This may suggest that additional mechanisms, such as inhibition of structural plasticity or epigenetic regulation of transcription, do limit the maximal potential for adult plasticity after all.

Alternatively, the maturation of inhibitory input does not only alter the levels of inhibition (or the balance between inhibition and excitation) but also causes qualitative changes in inhibitory synaptic transmission. Several recent findings seem to support this notion. One study has shown that when inhibitory neurons are isolated from 12–16-day-old embryo's and transplanted into newborn mice, a second sensitive period occurs around the time that the transplanted interneurons reached an equivalent of approximately 4 weeks postnatally, exactly when the sensitive period normally occurs in mice [46]. It did not matter whether the recipient mice were around 1 or 10 days old when they received the transplanted neurons, the age of the donor cells defined the onset of the second sensitive period. It seems unlikely that these inhibitory neurons reduce cortical inhibition around 5 weeks after transplantation unless they temporarily and specifically innervate other inhibitory neurons. More likely, inputs of immature inhibitory neurons at a restricted timepoint of development have particular properties necessary for sensitive period plasticity to occur. It is unclear what properties of immature inhibitory synapses would enhance cortical plasticity, but interesting possibilities are their reduced speed and increased short- and long-term plasticity potential. This is supported by two recent studies investigating the involvement of cannabinoid receptor (CB1R) mediated long-term depression of inhibitory inputs (iLTD) in sensitive period plasticity [47, 48]. These studies show that before and during the sensitive period, iLTD can be induced in layer 2/3 of the visual cortex and that this is probably mediated through CB1Rs present on inhibitory presynaptic terminals. In adult visual cortex, iLTD cannot be induced and inhibitory terminals are insensitive to CB1R agonist treatment. In parallel, inhibitory synapses show a reduction in short term depression with age. It was found that these changes do not occur in CB1R deficient animals and can be prevented by dark rearing animals into adulthood. These results indicate that CB1R mediated iLTD results in the maturation of inhibitory synapses, causing them to reduce GABA release probability but making them more suitable for fast transmission with less depression. These changes not only occurred in inhibitory synapses formed by cholecystokinin-positive interneurons which are known to express CB1R, but also in those formed by PV basket cells. Interestingly, there was a correlation between the inducibility of iLTD and OD plasticity. When adult mice were housed in the dark for two weeks, inhibitory synapses were found to rejuvenate and become sensitive to CB1R mediated depression again. At the same time, OD plasticity could be induced again in these animals. Both events could be prevented by treating the animals housed in the dark with a CB1R agonist or diazepam. These data thus support the notion that differences in the modifiability or and/or speed of immature inhibitory inputs makes them more suitable for allowing cortical plasticity to occur. While based on these studies it is tempting to speculate that it is the changes in inhibitory synapses of PV basket cells that are responsible for the changes in OD plasticity in the latter study, it cannot yet be excluded that CB1Rs present on excitatory or other inhibitory neurons are involved in this paradigm. This caveat actually holds true for quite a number of the studies discussed above. Thus, while several studies have now convincingly shown that PV basket cells are capable of initiating or reinitiating a sensitive period and play a crucial role in this, they do not exclude the possibility that other interneuron subsets also play an important role in cortical plasticity. It is important to keep this in mind when addressing the question how changes in inhibitory inputs permit or drive cortical plasticity.

## 7. Mechanisms by Which Inhibition May Alter Cortical Plasticity

**Figure 2:**
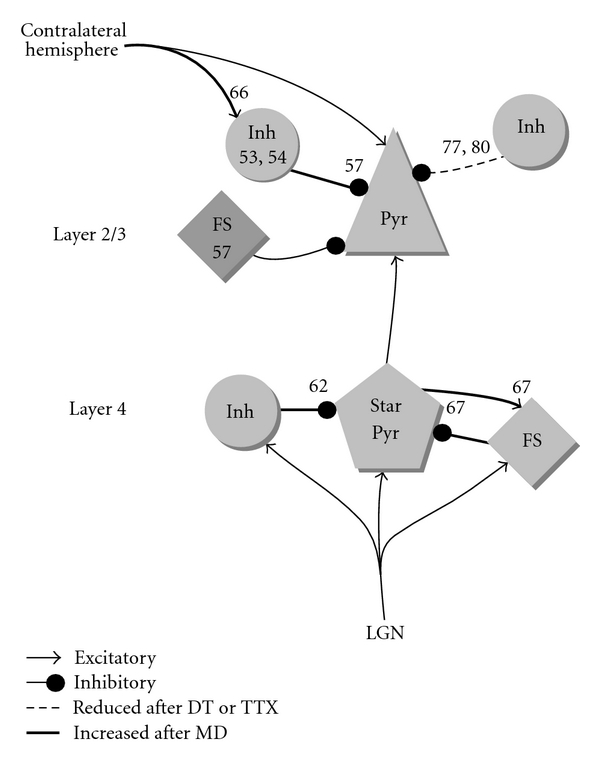
Documented changes in inhibition in V1 after monocular deprivation (MD), dark treatment (DT) or intraocular TTX injection during the critical period. Numbers correspond to the references that measured the change in the responses or synaptic strength of interneurons. Pyr is pyramidal cell, Star pyr is star pyramid neuron, Inh is interneuron, FS is fast-spiking interneuron. Light gray means shifted towards the open eye after MD. Dark gray is shifted towards the deprived-eye. All studies were done in binocular visual cortex, except for references [67, 77] which were done in monocular cortex and possibly reference [80], which left the exact location within visual cortex unspecified.

While evidence for maturation of inhibitory innervation regulating sensitive period onset is very solid, the mechanism of how inhibition regulates plasticity remains unclear. There are two likely explanations, which are not mutually exclusive. The first is that maturation of inhibition moderates the level of plasticity in excitatory synapses. It is known from slice physiology that reduced GABAergic inhibition facilitates synaptic plasticity [49]. PV basket cells, in particular, may alter plasticity, because they provide perisomatic inhibition which can effectively reduce action potential generation and dendritic back propagation. The second is that inhibition directly contributes to the expression of the OD shift. This was first suggested when intravenous injections of the GABA(A) antagonist bicuculline restored binocular responses in more than half of the neurons in visual cortex of amblyopic cats [50] (although a later study employing intracortical bicuculline application only found relatively weak effects on 29% of the neurons [51]). There is good evidence for such a mechanism during plasticity of auditory maps in the inferior colliculus of the barn owl [52]. When barn owls are reared wearing a prism, the maps for where auditory cues are located in visual space are altered. Interestingly, the old maps remain present but are suppressed by inhibition. Whether the same occurs in the visual cortex is still under intense investigation and up to now the results are equivocal. A graphical summary of the results is shown in Figure 2.

## 8. OD Plasticity in Interneurons

Suppression of deprived-eye responses by inhibition can in principle be achieved through increased excitation of interneurons responding to the deprived-eye, or through strengthening of their inputs onto excitatory neurons. A few studies have been published in recent years addressing these possibilities. We will first discuss three studies which have investigated how the responses of interneurons change during OD plasticity. Two of these employed two photon imaging of calcium responses of individual neurons in mice expressing a fluorescent protein in inhibitory neurons. The first study employed mice in which the GFP coding sequence was knocked into the *Gad-1 *gene (encoding the rate limiting glutamate decarboxylase GAD67) resulting in expression of GFP in most interneurons [53]. This study found that interneurons shift their responses towards the undeprived-eye, but in contrast to excitatory neurons not during the first days. This initial dysbalance of inhibition and excitation is expected to result in a stronger expression of the OD shift, but does not support the idea that OD plasticity is initiated by active suppression of deprived-eye responses through increased inhibition. The delay in the shift of interneurons may make plasticity of excitatory connections towards the open eye easier as initially they do not receive more inhibition. In a second study [54], using a transgenic mouse line in which a GFP variant (Venus) was expressed under the Vesicular GABA transporter (VGAT) promoter, it was found that interneurons and excitatory neurons shift to the same extent both during short-time deprivation and long-term deprivation. This finding thus partially contradicts the first study. It seems to be in line with the idea that responses of interneurons reflect the pooled activity of the surrounding population, as interneurons were also more binocular than excitatory neurons. When OD plasticity was induced in adult mice, it was found that interneurons show a larger shift than excitatory neurons, possibly restricting adult plasticity. It is interesting to speculate that this may explain why administering barbiturates or benzodiazepines, drugs which both enhance inhibition, reduces the measured OD shift in adult mice [55, 56]. The third study employed in vivo intracellular recordings to measure visual responses and identify fast-spiking basket cells [57]. Again, a different result was obtained. Fast-spiking interneurons showed a paradoxical shift towards the closed eye after two days of deprivation, while prolonged MD resulted in a similar shift of interneurons and excitatory neurons. This result is in line with the idea that the initial loss of responsiveness to deprived-eye inputs may be mediated by increased inhibition [58]. Unfortunately, we have to conclude that these studies provide contradictory results on how interneurons alter their responsiveness during the first days of OD plasticity and only agree that after prolonged MD both excitatory and inhibitory neurons shift their responsiveness towards the nondeprived-eye. This suggests that suppression of deprived-eye responses through inhibition is not the mechanism underlying the OD shift induced by long-term MD. Whether early plasticity of inhibitory neurons is important for initiating or facilitating OD plasticity remains unanswered, due to the contradictory results. It is therefore important to understand the reason for these different observations. The most likely explanations include differences in the subsets of interneurons that were assessed and technical issues that complicated the experiments.

Which interneuron subsets were assessed in the different studies? The two-photon studies employed different mouse lines in which GFP variants were expressed in interneurons. Although in the two mouse lines, there is good co-expression of GFP and GABA (in GAD67-GFP mice, 92% of all GFP+ cells are GABA+, while 96% of all GABA+ cells are GFP+ [59], in VGAT-Venus mice, 96% of Venus+ cells expressed GABA, while 93% of all GABA+ cells expressed Venus [60]), it is unclear whether expression levels of GFP vary per interneuron subtype. If so, subtypes of interneurons with lower expression levels of GFP may have been missed in the deeper layers where GFP detectability is lower with two-photon microscopy. In the study employing in vivo intracellular recordings, fast-spiking interneurons were selected based on their firing properties. This subset of interneurons represents approximately 10% of inhibitory neurons in layer 1, 60% in layer II, and 35% in layer III [59]. It may thus be that different types of interneurons were sampled and that these show different levels and signs of plasticity. A more complicated issue is that in GAD67-GFP mice, especially during the first weeks of development, GABA levels are reduced by 30–40% due to the inactivation of one *Gad-1* allele [61]. This may cause a delayed onset of the sensitive period and differences in the connectivity of interneurons [34] and hence affect interneuron plasticity induced by MD. More studies are thus necessary to learn how different interneuron subsets change their responsiveness with MD and what the consequences are for plasticity and expression of the OD shift.

## 9. Plasticity of Inhibitory Synapses

The above changes in interneuron responses will alter the inhibitory input to excitatory neurons. This input may also change by plasticity of inhibitory synapses themselves. Direct evidence that in the visual cortex these synapses are altered by MD comes from experiments in rats. During the sensitive period, two days of MD leads to an increase in the amplitude of GABA(A) miniature inhibitory postsynaptic currents (mIPSCs) in layer 4 star pyramidal neurons of the binocular visual cortex, without a change in mIPSC frequency [62]. After three days of deprivation, the amplitude nearly doubles and a large increase is still present after a week of MD. This suggests an increase in the strength of the GABA(A) inhibitory synapses onto these layer 4 excitatory neurons without a change in synapse numbers, which lasts throughout the period of deprivation. Interestingly, housing the animals in the dark for a week does not produce any change in the mIPSC amplitudes, suggesting that the changes in GABAergic synapses are dependent on input from the closed eye in response to low spatial frequency stimuli which can still be detected through the sutured eyelid, or on input from the open eye.

Although it is unknown if the synaptic changes induced by MD correlate with OD plasticity, it is likely that the increased inhibition reduces the responses of the deprived-eye. To prove this, it would be necessary to study the inhibitory synaptic strength in vivo and correlate it with OD. This experiment has not been done yet, but one study looked at the inhibitory contribution to the change in OD of pyramidal cells in vivo [57]. The OD in spike rate was measured before and after blocking GABA(A) receptors intracellularly. In control mice, during the sensitive period, the block of inhibition reduced the bias of pyramidal cell responses for one of the eyes. At first glance, this suggests that the spike bias of a pyramidal cell towards input of one eye is in part caused by preferential inhibition towards input of the other eye. This is not necessarily the case however, because also unbiased inhibition can increase the relative difference between the responses to the two eyes simply by reducing the absolute response strengths by an equal amount [63].

After MD, the removal of inhibition caused a reversal of the eye preference in the spike response. Such a reversal can only happen if the inhibitory input is biased in the same direction as the excitatory input, and opposite to the pyramidal cell's spiking output. The difference in the effect of removal of inhibition with and without MD can be interpreted as an increase in inhibition from deprived-eye stimulation [57, 64]. It is primarily, however, indicative of a relative change in strength of the inhibitory and excitatory biases, which could as well be caused by changes in excitation. It does show that after MD in the juvenile animal, there are cells where the preferred eye of their excitatory input is different from the preferred eye in their output. 

In the adult animal, no change in the spike biases was observed after removal of inhibition, even after MD. This means that in the adult animal the spiking bias is more similar to the excitatory input bias than in the juvenile animal. But even in adult animals, OD of the population response can be changed by altering inhibition by pharmacology, for instance in undeprived cats [65] and in monocularly deprived mice [55, 56].

More evidence suggesting that inhibition can alter the expression of the OD shift after MD comes from experiments assessing the influence of callosal connections on sensitive period plasticity. After a week of MD, the OD shift in the binocular visual cortex contralateral to the deprived-eye is removed by acutely silencing the visual cortex on the opposite side [66]. Surprisingly, this effect is not due to a reduction of the open eye responses, which dominate the silenced side of the cortex, but by a large increase in the deprived-eye responses after silencing. In undeprived animals, silencing contralateral cortex only produces the expected reduction in ipsilateral eye responses. The period of MD must cause an increase in inhibition mediated by callosal projections. It is likely that this increase is relayed by local interneurons, as only about one percent of the callossally projecting neurons are interneurons [66]. That contralateral silencing only affects deprived-eye responses could be because of a lack of increase in callosum-mediated deprived-eye excitation or a specific increase in deprived-eye inhibition. Calcium imaging studies, however, have not reported a shift towards the deprived-eye in interneurons [53, 54]. It could be that a specific subset of interneurons receiving callosal inputs has been missed. Alternatively, the incoming callosal synapses, and thus the spiking responses of the interneurons may not have changed, but their efferent synapses may have gained in inhibitory strength. 

This change in inhibitory synaptic strength, the reported increase in mini IPSC amplitude [62] and the reversed biases by blockade of inhibition [57] could all be explained if MD increases the strength of inhibitory synapses onto pyramidal cells. Such an increase could be similar to the potentiation of inhibition from fast-spiking cells onto star pyramids that occurs in monocular visual cortex after three days of deprivation just before the sensitive period [67]. 

Another interesting possible mechanism for the increase in synaptic strength of specifically deprived-eye dominated inhibitory synapses, is the possible reduction of the endocannabinoid receptor dependent form of long-term depression of inhibitory synapses (iLTD) [68] by MD. Inhibitory synapses may be partially depressed by normal vision during the sensitive period [69]. Perhaps MD prevents this depression in the synapses coming from interneurons that are dominated by the deprived-eye. This would result in a relative increase in inhibition caused by deprived-eye stimulation compared to open eye stimulation.

## 10. Inhibition and Homeostasis

Recently, it was discovered that MD does not reduce responses to the deprived-eye in all neurons in visual cortex, but that a selection of cells with little input from the open eye often show more response to the deprived-eye than in the normal situation [70]. This can be interpreted as a homeostatic reaction [71] to keep neural activity within an optimal range. The same homeostatic mechanism might be responsible for the increase normally seen in open eye responses after MD. Indeed, in mice deficient for TNF-alpha, a protein necessary for homeostatic scaling of excitatory and inhibitory synapses [72], the increase in open eye response does not occur [73]. This poses the question whether homeostasis of inhibition plays a role in OD plasticity. 

It has long been known that removal of visual input to the adult visual cortex leads to a decrease in levels of GABA, its synthesizing enzymes GAD65 and GAD67 and the beta2/3 subunit of the GABA(A) receptor [74–76]. Ten days of dark exposure also leads to a, possibly homeostatic, reduction of the amplitudes of IPSCs in rat layer 2/3 pyramidal cells evoked by layer 4 stimulation [47]. Similarly, intraocular TTX injection results two days later in reduced amplitudes of spontaneous IPSCs in rat layer 2/3 pyramidal neurons in the contralateral monocular cortex [77]. The opposing homeostatic action of increasing inhibition in response to raised activity levels, has also been found in culture and in vivo in the hippocampus after kainate injections [78] and in barrel cortex after whisker hyperstimulation [79]. A clear example of bidirectional homeostasis of inhibition was found in the mouse visual cortex at the start of the sensitive period. Similar to the results of rat adult dark treatment, two days of dark treatment reduced the frequency of miniature IPSCs in layer 2/3 pyramidal cells [80]. Two hours of light, and thus visual input, after the dark treatment immediately lead to an increase in amplitude and frequency of mIPSCs, suggesting an increase of GABAergic input in response to the increase in cortical activity. In rat star pyramidal cells in layer 4, however, a longer period of 7–17 days of dark treatment during the sensitive period did not change mIPSCs [62]. These results suggest that the occurrence and timing of homeostasis is layer or species dependent. This is not surprising, because homeostatic effects are also cell type specific [81] and even strain dependent, as different mouse strains showed different amounts of potentiation of open eye responses [82]. It is still unclear if a homeostatic response of inhibitory connections in binocular visual cortex is at all induced by MD during the sensitive period. In contrast to studies of adult MD, the distribution of GAD65 labeling in cat layer 4 does not change after two or seven days of MD [83]. On average, star pyramids in the same layer of rat binocular V1 even show a large increase in mIPSC amplitudes in response to MD [62]. The loss of input may thus lead to an increase in inhibition, which is contrary to what one would expect of an homeostatic response. Without categorization by eye preference of the postsynaptic neurons, however, homeostatic changes may be masked by opposite changes induced by the competition between open and closed eye inputs. It therefore remains possible that homeostasis of inhibition plays a role in OD plasticity during the sensitive period, but unambiguous evidence for such a role is still lacking.

## 11. Inhibition Setting the Threshold for Excitatory Plasticity

As discussed earlier, the state of the inhibitory system is an important factor in the opening of the sensitive period. This correlation between the state of inhibition and plasticity may be through changes in the plasticity of the response or connections of inhibitory neurons themselves, as suggested for instance by the experiments on iLTD [47]. Alternatively, it may be that the prime influence of inhibition on OD plasticity is by gating excitatory transmission of information [84] or setting the level of excitatory plasticity. It is well established that blockade of inhibition facilitates LTP induction [49]. Reducing inhibition for a week by cortical infusion of an GABAAR antagonist even reinstates white matter to layer 2/3 LTP in the adult visual cortex [45]. Blocking GABAergic inhibition when pairing presynaptic activity with single postsynaptic action potentials also facilitates spike timing-dependent LTP [85]. Inhibition impinging on the soma of pyramidal cells (such as that coming from fast-spiking PV basket cells) is particularly well placed to influence plasticity throughout a neuron by preventing action potential generation [86] or back-propagation [87] by hyperpolarization or shunting. And while reduction of inhibition thus possibly facilitates potentiation of excitatory connections dominated by open eye input, an increase in inhibition has been shown to induce the reduction of these connections [88].

A more indirect way through which inhibition may alter plasticity is through controlling brain oscillations. In particular fast-spiking PV basket cells are important for the generation of gamma rhythms in the brain [26, 89–91]. The presence of oscillations may lead to more efficient transfer of information across the cortex and contribute to coincident spiking, which in turn may increase the occurrence of spike timing-dependent plasticity.

## 12. Outlook

During the last decade convincing evidence has accumulated showing that the development of inhibitory synapses formed by PV interneurons defines the onset of the sensitive period of OD plasticity in V1. However, we are still in the dark about the mechanisms by which inhibitory innervation permits or instructs these experience dependent changes in neuronal responsiveness. A crucial question that remains unanswered is whether PV basket cells permit cortical plasticity by influencing neuronal synchrony and spike timing-dependent plasticity, or instruct plasticity by specifically suppressing deprived-eye responses. Experiments to address these issues will be technically difficult. Studying whether spike timing-dependent plasticity is altered through the changing influence of PV basket cell inputs during development would require in vivo experiments in which electric stimulation of individual pyramidal neurons is time-locked to visually evoked responses, a challenging undertaking. To test whether plasticity of inhibitory inputs influence the expression of OD or its plasticity, experiments involving the inactivation of interneuron plasticity or brief suppression of specific interneuron subsets during the assessment of OD will be required.

There is also an almost complete lack of information on the dynamics of structural plasticity of excitatory inputs onto inhibitory neurons and vice versa during OD plasticity. Knowledge is currently limited to the finding that there is an increase in growth and retraction of inhibitory dendritic branch tips upon MD in early adulthood [92]. This suggests that inhibitory neurons alter their inputs during plasticity, possibly in line with an initial shift of interneurons away from the deprived-eye as observed by Kameyama et al. [54] or with a homeostatic decrease of inhibition with reduced input. More direct methods for visualizing excitatory synapse onto interneurons and inhibitory synapses onto excitatory neurons in vivo would be required to address this important issue.

Although until now evidence points towards PV basket cells being important for initiating the sensitive period, they are most likely not the only subset of interneurons that is involved in cortical plasticity. We will therefore need to dissect which types of interneurons are involved in any of the mechanisms described above. This will also require better insight into the exact wiring diagrams of excitatory and inhibitory neurons within and between different cortical layers. The production of mice expressing the Crerecombinase in specific interneurons subsets will be of tremendous help to achieve this.

A recent study has shown that there are important differences between the development of GABAergic innervation in layer 4 and layers 2/3 [69]. It was found that in layer 4, inhibitory synapse development occurs approximately one week earlier, is much less sensitive to dark rearing, and is not sensitive to endocannabinoids. Previous studies have shown that the different cortical layers can undergo OD plasticity with a surprising level of independence [93, 94]. Moreover, it was found that the expression of OD in layer 4 is not influenced by inhibition [95]. These finding may indicate that the role of inhibitory innervation in sensitive period onset and offset may be restricted to the pyramidal layers. If so, this will have important consequences for how successful strategies to reactivate cortical plasticity based on altering inhibition will be in high-acuity animals with a columnar organization of the visual cortex. In such species, thalamocortical projections show more extensive retractions after MD than in rodents [8, 96]. If plasticity in layer 4 remains limited upon manipulating inhibitory innervation in adult animals, recovery of vision may be rather limited in contrast to what is observed in rodents. If we want to fulfill the promise of developing treatments for neurodevelopmental disorders and recovery of defective brain function due to physical trauma or plasticity gone wrong, we will need to address these open questions so that highly specific and effective approaches can be developed for reactivating plasticity in the adult neocortex.

## References

[1] Morishita H, Hensch TK (2008). Critical period revisited: impact on vision. *Current Opinion in Neurobiology*.

[2] Stiles J (2000). Neural plasticity and cognitive development. *Developmental Neuropsychology*.

[3] Wiesel TN, Hubel DH (1963). Single-Cell responses in striate cortex of kittens deprived of vision in one eye. *Journal of Neurophysiology*.

[4] Ito M (2004). ’Nurturing the brain’ as an emerging research field involving child neurology. *Brain and Development*.

[5] Hubel DH, Wiesel TN (1962). Receptive fields, binocular interaction and functional architecture in the cat’s visual cortex. *The Journal of physiology*.

[6] Levi D (2006). Visual processing in amblyopia: human studies. *Strabismus*.

[7] Hubel DH, Wiesel TN, LeVay S (1977). Plasticity of ocular dominance columns in monkey striate cortex. *Philosophical Transactions of the Royal Society of London B*.

[8] Antonini A, Fagiolini M, Stryker MP (1999). Anatomical correlates of functional plasticity in mouse visual cortex. *Journal of Neuroscience*.

[9] Antonini A, Stryker MP (1996). Plasticity of geniculocortical afferents following brief or prolonged monocular occlusion in the cat. *Journal of Comparative Neurology*.

[10] Trachtenberg JT, Stryker MP (2001). Rapid anatomical plasticity of horizontal connections in the developing visual cortex. *Journal of Neuroscience*.

[11] Gordon JA, Stryker MP (1996). Experience-dependent plasticity of binocular responses in the primary visual cortex of the mouse. *Journal of Neuroscience*.

[12] Fagiolini M, Pizzorusso T, Berardi N, Domenici L, Maffei L (1994). Functional postnatal development of the rat primary visual cortex and the role of visual experience: dark rearing and monocular deprivation. *Vision Research*.

[13] Jones KR, Spear PD, Tong L (1984). Critical periods for effects of monocular deprivation: differences between striate and extrastriate cortex. *Journal of Neuroscience*.

[14] Vaegan, Taylor D (1979). Critical period for deprivation amblyopia in children. *Transactions of the Ophthalmological Societies of the United Kingdom*.

[15] Hensch TK, Fagiolini M, Mataga N, Stryker MP, Baekkeskov S, Kash SF (1998). Local GABA circuit control of experience-dependent plasticity in developing visual cortex. *Science*.

[16] Pizzorusso T, Medini P, Berardi N, Chierzi S, Fawcett JW, Maffei L (2002). Reactivation of ocular dominance plasticity in the adult visual cortex. *Science*.

[17] Putignano E, Lonetti G, Cancedda L (2007). Developmental Downregulation of Histone Posttranslational Modifications Regulates Visual Cortical Plasticity. *Neuron*.

[18] Fagiolini M, Hensch TK (2000). Inhibitory threshold for critical-period activation in primary visual cortex. *Nature*.

[19] Huang ZJ, Kirkwood A, Pizzorusso T (1999). BDNF regulates the maturation of inhibition and the critical period of plasticity in mouse visual cortex. *Cell*.

[20] Hanover JL, Huang ZJ, Tonegawa S, Stryker MP (1999). Brain-derived neurotrophic factor overexpression induces precocious critical period in mouse visual cortex. *The Journal of Neuroscience*.

[21] Zafra F, Hengerer B, Leibrock J, Thoenen H, Lindholm D (1990). Activity dependent regulation of BDNF and NGF mRNAs in the rat hippocampus is mediated by non-NMDA glutamate receptors. *EMBO Journal*.

[22] Gianfranceschi L, Siciliano R, Walls J (2003). Visual cortex is rescued from the effects of dark rearing by overexpression of BDNF. *Proceedings of the National Academy of Sciences of the United States of America*.

[23] Ciucci F, Putignano E, Baroncelli L, Landi S, Berardi N, Maffei L (2007). Insulin-like growth factor 1 (IGF-1) mediates the effects of enriched environment (EE) on visual cortical development. *PLoS ONE*.

[24] Carro E, Nuñez A, Busiguina S, Torres-Aleman I (2000). Circulating insulin-like growth factor I mediates effects of exercise on the brain. *Journal of Neuroscience*.

[25] Tamás G, Buhl EH, Somogyi P (1997). Fast IPSPs elicited via multiple synaptic release sites by different types of GABAergic neurone in the cat visual cortex. *Journal of Physiology*.

[26] Galarreta M, Hestrin S (2002). Electrical and chemical synapses among parvalbumin fast-spiking GABAergic interneurons in adult mouse neocortex. *Proceedings of the National Academy of Sciences of the United States of America*.

[27] Kawaguchi Y, Kubota Y (1993). Correlation of physiological subgroupings of nonpyramidal cells with parvalbumin- and calbindin(D28k)-immunoreactive neurons in layer V of rat frontal cortex. *Journal of Neurophysiology*.

[28] Klausberger T, Roberts JDB, Somogyi P (2002). Cell Type- and Input-Specific Differences in the Number and Subtypes of Synaptic GABAA Receptors in the Hippocampus. *Journal of Neuroscience*.

[29] Brussaard AB, Kits KS, Baker RE (1997). Plasticity in fast synaptic inhibition of adult oxytocin neurons caused by switch in GABA(A) receptor subunit expression. *Neuron*.

[30] Fagiolini M, Fritschy JM, Löw K, Möhler H, Rudolph U, Hensch TK (2004). Specific GABAA Circuits for Visual Cortical Plasticity. *Science*.

[31] Baude A, Bleasdale C, Dalezios Y, Somogyi P, Klausberger T (2007). Immunoreactivity for the GABAA receptor alpha1 subunit, somatostatin and Connexin36 distinguishes axoaxonic, basket, and bistratified interneurons of the rat hippocampus. *Cerebral Cortex*.

[32] Sugiyama S, Di Nardo AA, Aizawa S (2008). Experience-Dependent Transfer of Otx2 Homeoprotein into the Visual Cortex Activates Postnatal Plasticity. *Cell*.

[33] Di Cristo G, Wu C, Chattopadhyaya B (2004). Subcellular domain-restricted GABAergic innervation in primary visual cortex in the absence of sensory and thalamic inputs. *Nature Neuroscience*.

[34] Chattopadhyaya B, Di Cristo G, Wu CZ (2007). GAD67-Mediated GABA Synthesis and Signaling Regulate Inhibitory Synaptic Innervation in the Visual Cortex. *Neuron*.

[35] Di Cristo G, Chattopadhyaya B, Kuhlman SJ (2007). Activity-dependent PSA expression regulates inhibitory maturation and onset of critical period plasticity. *Nature Neuroscience*.

[36] McGee AW, Yang Y, Fischer QS, Daw NW, Strittmatter SH (2005). Neuroscience: experience-driven plasticity of visual cortex limited by myelin and nogo receptor. *Science*.

[37] Bradbury EJ, Moon LDF, Popat RJ (2002). Chondroitinase ABC promotes functional recovery after spinal cord injury. *Nature*.

[38] De Wit J, Verhaagen J (2007). Proteoglycans as modulators of axon guidance cue function. *Advances in Experimental Medicine and Biology*.

[39] Oray S, Majewska A, Sur M (2004). Dendritic spine dynamics are regulated by monocular deprivation and extracellular matrix degradation. *Neuron*.

[40] Mataga N, Nagai N, Hensch TK (2002). Permissive proteolytic activity for visual cortical plasticity. *Proceedings of the National Academy of Sciences of the United States of America*.

[41] Carulli D, Pizzorusso T, Kwok JCF (2010). Animals lacking link protein have attenuated perineuronal nets and persistent plasticity. *Brain*.

[42] Luth HJ, Fischer J, Celio MR (1992). Soybean lectin binding neurons in the visual cortex of the rat contain parvalbumin and are covered by glial nets. *Journal of Neurocytology*.

[43] Sale A, Maya Vetencourt JF, Medini P (2007). Environmental enrichment in adulthood promotes amblyopia recovery through a reduction of intracortical inhibition. *Nature Neuroscience*.

[44] Maya Vetencourt JF, Sale A, Viegi A (2008). The antidepressant fluoxetine restores plasticity in the adult visual cortex. *Science*.

[45] Harauzov A, Spolidoro M, DiCristo G (2010). Reducing intracortical inhibition in the adult visual cortex promotes ocular dominance plasticity. *Journal of Neuroscience*.

[46] Southwell DG, Froemke RC, Alvarez-Buylla A, Stryker MP, Gandhi SP (2010). Cortical plasticity induced by inhibitory neuron transplantation. *Science*.

[47] Huang S, Gu Y, Quinlan EM, Kirkwood A (2010). A refractory period for rejuvenating GABAergic synaptic transmission and ocular dominance plasticity with dark exposure. *Journal of Neuroscience*.

[48] Jiang B, Huang S, de Pasquale R (2010). The maturation of GABAergic transmission in visual cortex requires endocannabinoid-mediated LTD of inhibitory inputs during a critical period. *Neuron*.

[49] Wigstrom H, Gustafsson B (1983). Facilitated induction of hippocampal long-lasting potentiation during blockade of inhibition. *Nature*.

[50] Duffy FH, Snodgrass SR, Burchfiel JL, Conway JL (1976). Bicuculline reversal of deprivation amblyopia in the cat. *Nature*.

[51] Sillito AM, Kemp JA, Blakemore C (1981). The role of GABAergic inhibition in the cortical effects of monocular deprivation. *Nature*.

[52] Zheng W, Knudsen EI (1999). Functional selection of adaptive auditory space map by GABA(a)-mediated inhibition. *Science*.

[53] Gandhi SP, Yanagawa Y, Stryker MP (2008). Delayed plasticity of inhibitory neurons in developing visual cortex. *Proceedings of the National Academy of Sciences of the United States of America*.

[54] Kameyama K, Sohya K, Ebina T, Fukuda A, Yanagawa Y, Tsumoto T (2010). Difference in binocularity and ocular dominance plasticity between GABAergic and excitatory cortical neurons. *Journal of Neuroscience*.

[55] Pham TA, Graham SJ, Suzuki S (2004). A semi-persistent adult ocular dominance plasticity in visual cortex is stabilized by activated CREB. *Learning and Memory*.

[56] Heimel JA, Hartman RJ, Hermans JM, Levelt CN (2007). Screening mouse vision with intrinsic signal optical imaging. *European Journal of Neuroscience*.

[57] Yazaki-Sugiyama Y, Kang S, Cteau H, Fukai T, Hensch TK (2009). Bidirectional plasticity in fast-spiking GABA circuits by visual experience. *Nature*.

[58] Kerlin AM, Andermann ML, Berezovskii VK, Reid RC (2010). Broadly Tuned Response Properties of Diverse Inhibitory Neuron Subtypes in Mouse Visual Cortex. *Neuron*.

[59] Young A, Sun QQ (2009). GAB aergic inhibitory interneurons in the posterior piriform cortex of the GAD67-GFP mouse. *Cerebral Cortex*.

[60] Wang Y, Kakizaki T, Sakagami H (2009). Fluorescent labeling of both GABAergic and glycinergic neurons in vesicular GABA transporter (VGAT)-Venus transgenic mouse. *Neuroscience*.

[61] Tamamaki N, Yanagawa Y, Tomioka R, Miyazaki JI, Obata K, Kaneko T (2003). Green Fluorescent Protein Expression and Colocalization with Calretinin, Parvalbumin, and Somatostatin in the GAD67-GFP Knock-In Mouse. *Journal of Comparative Neurology*.

[62] Maffei A, Lambo ME, Turrigiano GG (2010). Critical period for inhibitory plasticity in rodent binocular V1. *Journal of Neuroscience*.

[63] Priebe NJ (2008). The relationship between subthreshold and suprathreshold ocular dominance in cat primary visual cortex. *Journal of Neuroscience*.

[64] Smith GB, Bear MF (2010). Bidirectional ocular dominance plasticity of inhibitory networks: recent advances and unresolved questions. *Frontiers in Cellular Neuroscience*.

[65] Sillito AM, Kemp JA, Patel H (1980). Inhibitory interactions contributing to the ocular dominance of monocularly dominated cells in the normal cat striate cortex. *Experimental Brain Research*.

[66] Restani L, Cerri C, Pietrasanta M, Gianfranceschi L, Maffei L, Caleo M (2009). Functional masking of deprived eye responses by callosal input during ocular dominance plasticity. *Neuron*.

[67] Maffei A, Nataraj K, Nelson SB, Turrigiano GG (2006). Potentiation of cortical inhibition by visual deprivation. *Nature*.

[68] Chevaleyre V, Castillo PE (2004). Endocannabinoid-mediated metaplasticity in the hippocampus. *Neuron*.

[69] Jiang B, Sohya K, Sarihi A, Yanagawa Y, Tsumoto T (2010). Laminar-specific maturation of GABAergic transmission and susceptibility to visual deprivation are related to endocannabinoid sensitivity in mouse visual cortex. *Journal of Neuroscience*.

[70] Mrsic-Flogel TD, Hofer SB, Ohki K, Reid RC, Bonhoeffer T, Hübener M (2007). Homeostatic regulation of eye-specific responses in visual cortex during ocular dominance plasticity. *Neuron*.

[71] Turrigiano GG, Nelson SB (2004). Homeostatic plasticity in the developing nervous system. *Nature Reviews Neuroscience*.

[72] Stellwagen D, Malenka RC (2006). Synaptic scaling mediated by glial TNF-alpha. *Nature*.

[73] Kaneko M, Stellwagen D, Malenka RC, Stryker MP (2008). Tumor necrosis factor-alpha mediates one component of competitive, experience-dependent plasticity in developing visual cortex. *Neuron*.

[74] Hendry SHC, Jones EG (1986). Reduction in numbers of immunostained GABAergic neurones in deprived-eye dominance columns of monkey area 17. *Nature*.

[75] Benevento LA, Bakkum BW, Cohen RS (1995). Gamma-aminobutyric acid and somatostatin immunoreactivity in the visual cortex of normal and dark-reared rats. *Brain Research*.

[76] He HY, Hodos W, Quinlan EM (2006). Visual deprivation reactivates rapid ocular dominance plasticity in adult visual cortex. *Journal of Neuroscience*.

[77] Maffei A, Turrigiano GG (2008). Multiple modes of network homeostasis in visual cortical layer 2/3. *Journal of Neuroscience*.

[78] Peng YR, Zeng SY, Song HL, Li MY, Yamada MK, Yu X (2010). Postsynaptic spiking homeostatically induces cell-autonomous regulation of inhibitory inputs via retrograde signaling. *Journal of Neuroscience*.

[79] Knott GW, Quairiaux C, Genoud C, Welker E (2002). Formation of dendritic spines with GABAergic synapses induced by whisker stimulation in adult mice. *Neuron*.

[80] Gao M, Sossa K, Song L (2010). A specific requirement of Arc/Arg3.1 for visual experience-induced homeostatic synaptic plasticity in mouse primary visual cortex. *Journal of Neuroscience*.

[81] Maffei A, Nelson SB, Turrigiano GG (2004). Selective reconfiguration of layer 4 visual cortical circuitry by visual deprivation. *Nature Neuroscience*.

[82] Heimel JA, Hermans JM, Sommeijer JP (2008). Genetic control of experience-dependent plasticity in the visual cortex. *Genes, Brain and Behavior*.

[83] Silver MA, Stryker MP (2000). Distributions of synaptic vesicle proteins and GAD65 in deprived and nondeprived ocular dominance columns in layer IV of kitten primary visual cortex are unaffected by monocular deprivation. *Journal of Comparative Neurology*.

[84] Rozas C, Frank H, Heynen AJ, Morales B, Bear MF, Kirkwood A (2001). Developmental inhibitory gate controls the relay of activity to the superficial layers of the visual cortex. *Journal of Neuroscience*.

[85] Meredith RM, Floyer-Lea AM, Paulsen O (2003). Maturation of long-term potentiation induction rules in rodent hippocampus: role of GABAergic inhibition. *Journal of Neuroscience*.

[86] Pouille F, Scanziani M (2001). Enforcement of temporal fidelity in pyramidal cells by somatic feed-forward inhibition. *Science*.

[87] Tsubokawa H, Ross WN (1996). IPSPs modulate spike backpropagation and associated [Ca2+](i) changes in the dendrites of hippocampal CA1 pyramidal neurons. *Journal of Neurophysiology*.

[88] Hata Y, Tsumoto T, Stryker MP (1999). Selective pruning of more active afferents when cat visual cortex is pharmacologically inhibited. *Neuron*.

[89] Sohal VS, Zhang F, Yizhar O, Deisseroth K (2009). Parvalbumin neurons and gamma rhythms enhance cortical circuit performance. *Nature*.

[90] Cardin JA, Carlén M, Meletis K (2009). Driving fast-spiking cells induces gamma rhythm and controls sensory responses. *Nature*.

[91] Tamás G, Buhl EH, Lörincz A, Somogyi P (2000). Proximally targeted GABAergic synapses and gap junctions synchronize cortical interneurons. *Nature Neuroscience*.

[92] Chen JL, Lin WC, Cha JW, So PT, Kubota Y, Nedivi E (2011). Structural basis for the role of inhibition in facilitating adult brain plasticity. *Nature Neuroscience*.

[93] Trachtenberg JT, Trepel C, Stryker MP (2000). Rapid extragranular plasticity in the absence of thalamocortical plasticity in the developing primary visual cortex. *Science*.

[94] Liu CH, Heynen AJ, Shuler MGH, Bear MF (2008). Cannabinoid receptor blockade reveals parallel plasticity mechanisms in different layers of mouse visual cortex. *Neuron*.

[95] Khibnik LA, Cho KKA, Bear MF (2010). Relative contribution of feedforward excitatory connections to expression of ocular dominance plasticity in layer 4 of visual cortex. *Neuron*.

[96] Antonini A, Gillespie DC, Crair MC, Stryker MP (1998). Morphology of single geniculocortical afferents and functional recovery of the visual cortex after reverse monocular deprivation in the kitten. *Journal of Neuroscience*.

